# Experts’ wisdom: challenges, rewards, key clinician characteristics, and advice in eating disorder treatment

**DOI:** 10.1186/s40337-025-01438-0

**Published:** 2025-10-27

**Authors:** Ivan Ejdemyr, Rasmus Isomaa, Kjersti Solhaug Gulliksen, Deborah Lynn Reas, Johanna Levallius

**Affiliations:** 1 Department of Clinical Sciences, Norrland University Hospital, Umea University, Umeå, 90185 Sweden; 2 National Specialized Medical Care for Eating Disorders, Region Vasternorrland, Sundsvall, Sweden; 3 Wellbeing Services County of Ostrobothnia, Fredrikakliniken, Jakobstad, Finland; 4 Faculty of Education & Welfare Studies, Abo Akademi University, Vasa, Finland; 5 The Norwegian Psychological Association & The Institute of Eating Disorders, Hunger House, Oslo, Norway; 6 Division of Mental Health and Addiction, Regional Department for Eating Disorders, Oslo University Hospital, Oslo, Norway

**Keywords:** Eating disorders, Professional competence, Attitude of health personnel, Professional–patient relations, Resilience, Workplace

## Abstract

**Background:**

Health professionals are crucial to the treatment of patients with eating disorders, and their experiential knowledge is central for bridging the gap between research and clinical practice. This study investigated why health professionals enter the eating disorder field, the challenges and rewards they face, the characteristics they consider vital for treating patients, and their advice to newcomers.

**Methods:**

Open-ended responses from 188 Nordic health professionals who work with eating disorders were collected via a cross-sectional online survey. Data were analyzed using quantitative and qualitative content analysis, and coding was conducted by multiple researchers using consensus procedures to enhance credibility and analytic rigor.

**Results:**

Participants reported entering the eating disorder field due to interest in the subject, by coincidence, or a desire to help. Primary challenges were patient ambivalence, building therapeutic relationships, and systemic constraints. The most rewarding aspects were patient recovery, facilitating change, and the therapeutic relationship. Patience and warmth were the most important professional characteristics, followed by maturity and expertise. Advice to new professionals emphasized adopting a therapeutic stance grounded in patience and curiosity, building specialized knowledge about eating disorders, and seeking support through supervision.

**Conclusions:**

These findings highlight the intertwined nature of challenges and rewards, and underscore the need to cultivate specific professional characteristics within supportive organizational contexts. By clarifying foundational conditions of day-to-day treatment, the results can inform workforce development, training, and healthcare service design to support retention and, in turn, high-quality treatment.

**Supplementary Information:**

The online version contains supplementary material available at 10.1186/s40337-025-01438-0.

## Abstract (plain language summary)

We asked health professionals who specialize in eating disorders why they started working in the field, what they find most challenging and most rewarding about the work, which professional qualities matter most for treating people with eating disorders, and what advice they would give to new colleagues. In total, 188 professionals across the Nordic countries answered the questions. Many said they began through personal interest, chance opportunities, or a wish to help. Common challenges included working with people who feel torn between wanting recovery and holding on to the eating disorder, the emotional demands of building trust, and limited time and resources in healthcare. At the same time, helping people make meaningful progress toward change was described as deeply rewarding; for example, seeing someone “return to life”. Patience, warmth, maturity, and specialist knowledge of eating disorders were highlighted as the most important professional qualities. The advice to newcomers was to use a patient and curious approach with each person, keep learning about eating disorders, and rely on supervision and teamwork. These insights point to practical steps for healthcare services, including investing in training and regular supervision, allow time for relationship-building, and support strong teams to help health professionals stay in the field and support high-quality care.

**Supplementary Information:**

The online version contains supplementary material available at 10.1186/s40337-025-01438-0.

## Introduction

At all levels of healthcare, health professionals play a crucial role in treating patients with eating disorders. Challenges for health professionals in the eating disorder field, along with suggested coping strategies used to address these challenges, are well-documented and remain an important area of research (e.g., [1, 2]). However, less attention has been paid to the rewards that sustain health professionals’ engagement and important characteristics they should cultivate. Given the complex clinical presentations and the need for specialized skills for health professionals in the eating disorder field [3], understanding these interconnected aspects is important for promoting sustainable employability, especially since high turnover rates in mental health services more broadly often exceed 50% and have been linked to compromised treatment quality (e.g., [4]). Achieving sustainability for health professionals requires balancing challenges with the rewards and resources that support them, and cultivating professional and organizational characteristics that foster long-term engagement [5].

When treating eating disorders, health professionals face numerous challenges at different, interconnected levels. At the individual level, health professionals sometimes struggle with limited knowledge, confidence, and high emotional demands, particularly when managing life-threatening behaviours and navigating uncertainty in the absence of clear evidence-based guidance [1, 6]. At the interpersonal level, patients with eating disorders present clinical challenges, commonly attributed to ambivalence, hesitancy around treatment, or fluctuating motivation, which can complicate therapeutic relationships [2, 7, 8]. At the organizational level, many healthcare settings, including specialized eating disorder units, are challenged by staffing shortages, resource constraints, and limited access to specialized multidisciplinary teams across both inpatient and outpatient settings [9, 10].

Given these challenges, understanding the rewards that professionals experience is important not only in its own right, but also in relation to what sustains long-term engagement in the field. Research into the rewards experienced by health professionals in the eating disorder field is limited. Existing studies, which predominantly focus on inpatient settings, indicate that health professionals find rewards in witnessing patients’ recovery and establishing therapeutic relationships [11, 12]. To our knowledge, however, rewards have rarely been systematically quantified or explored outside inpatient care, despite their importance for job satisfaction and retention [4].

In addition to challenges and rewards, the characteristics of health professionals influence how they approach their clinical work and play a critical role in treatment outcomes (e.g., [13]). However, major American and European treatment guidelines do not provide explicit recommendations regarding specific clinician characteristics in the eating disorder field; instead, they focus primarily on formal competence [3, 14]. In contrast, the Australia & New Zealand Academy for Eating Disorders has highlighted clinician characteristics such as firmness, empathy, expertise, and relational competence as particularly important [15]. Moreover, patient perspectives emphasize acceptance, vitality, challenge, and expertise as important clinician characteristics in eating disorder treatment [16].

Together, challenges, rewards, and clinician characteristics reflect interrelated dimensions of what it takes to sustain a workforce in eating disorder care. Understanding how professionals experience and navigate these dimensions—what strains them, what sustains them, and what they see as important characteristics—can inform strategies to support, train, and retain staff.

This study aims to investigate (1) why health professionals initially began working in the eating disorder field (2), the challenges and (3) rewards they encounter (4), key characteristics they consider important for working with eating disorders, and (5) advice for newcomers to the field. By combining qualitative and quantitative approaches with diverse professional perspectives, this study gives new and expanded evidence to help bridge the gap between research and clinical practice in the eating disorder field [17].

## Methods

This study used qualitative and quantitative content analysis of open-ended survey responses collected as part of a larger study on health professionals’ experiences working with patients with eating disorders. The study received ethical approval from the Departmental Institutional Review Board at the Institute of Psychology, Faculty of Social Sciences, University of Oslo (Reference No. 3485951). Participation was voluntary and anonymous, and electronic informed consent was obtained from all participants before they accessed the survey. No financial compensation was provided.

### Participants and setting

Participants were 188 health professionals attending the 2018 Nordic Eating Disorders Society (NEDS) meeting in Reykjavik, Iceland, and members of the associated national societies in Sweden, Denmark, Norway, Finland, and Iceland. NEDS is a professional forum uniting eating disorder health professionals in the Nordic countries to promote collaboration, research, and knowledge exchange, including through biennial conferences [18].

### Data collection

The health professionals were invited to take part in the online survey via email at the opening of the NEDS conference, through flyers distributed during the conference, and via the member mailing lists of the Nordic eating disorder societies. The survey remained open throughout the duration of the conference. After the conference, an email was sent to the Nordic societies with a request to distribute the survey link to their members to maximize reach and encourage participation. The conference had approximately 260 attendees, and the Nordic eating disorder societies collectively have around 1,000 active members. Data was collected using Nettskjema, an online survey platform managed by the University of Oslo. The survey included five open-ended questions:

1. What are some reasons you decided to work in the eating disorder field?
2. What do you find to be the most challenging aspect of working with eating disorders?
3. What do you find to be the most rewarding aspect of working with eating disorders?
4. What do you think are three important characteristics of a clinician who treats eating disorder patients?
5. What advice would you give to someone new working in the eating disorder field?

### Data analysis

For content analysis, we followed the guidelines by Erlingsson and Brysiewicz [19] and conducted manual, inductive coding. In line with this framework, we adopted a pragmatic, low-inference approach to content analysis. While primarily descriptive in nature, this approach acknowledges that interpretation is inherent to all qualitative analysis, and that researcher pre-understandings and contextual knowledge shape how meaning is constructed from the data. Participants’ responses to the open-ended questions could contain multiple meaning units, which were coded separately. Non-English answers were translated into English by the coders (IE, RI, JL) during analysis. Translations were cross-checked through coder consensus discussions; no formal back-translation was conducted.

The analytic process proceeded in several steps. First, we independently generated data-driven codes. Second, we grouped codes into patterns and relationships. Third, we developed categories and themes. Fourth, we documented codes, categories, and themes in a codebook. Subthemes were inductively derived from the coded data and grouped into higher-order categories and overarching themes. In some questions, themes were constructed directly from codes without subthemes, depending on the depth and complexity of the data. Coding was conducted by multiple researchers and finalized by consensus, with iterative codebook revisions to enhance credibility and analytic rigor. To describe salience, we computed code frequencies (counts); inferential statistics were not performed. While code counts were used descriptively to indicate salience, analytical significance was determined through iterative interpretation of patterns and meanings across responses, rather than frequency alone.

**Fig. 1:**
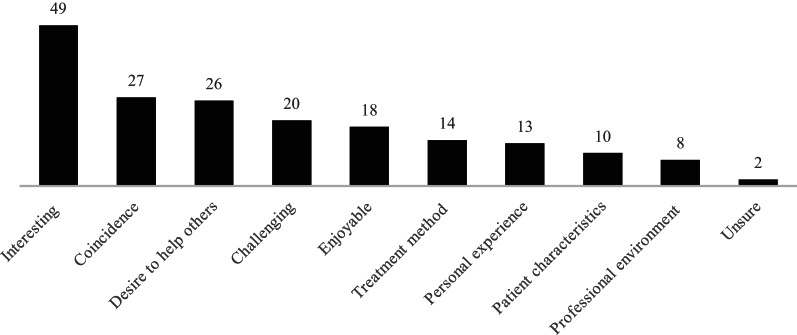
Reasons for beginning work in the field of eating disorders. Numbers on the bars indicate the number of coded responses (N = 135; 187 codes)

**Fig. 2:**
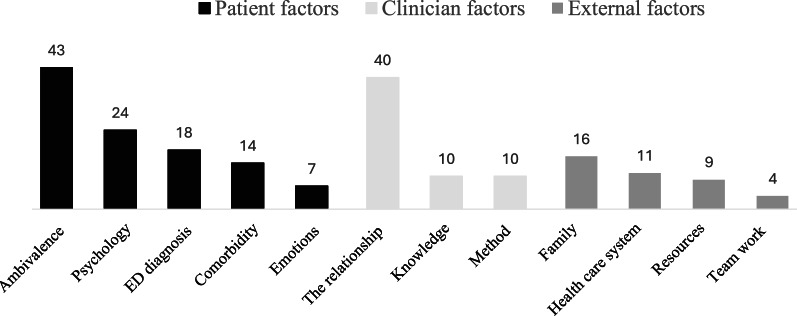
Challenges in working with eating disorders. Numbers on the bars (color-coded by theme) indicate the number of coded responses for each subtheme (N = 168; 206 codes). ED = eating disorder

**Fig. 3:**
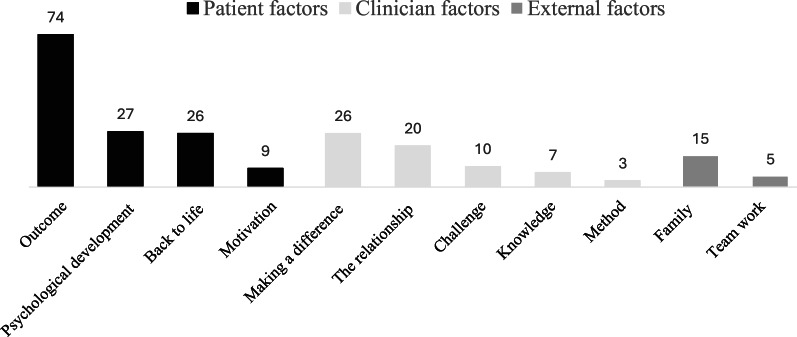
Rewards in working with eating disorders. Numbers on the bars (color-coded by theme) indicate the number of coded responses for each subtheme (N = 170; 222 codes).

**Fig. 4:**
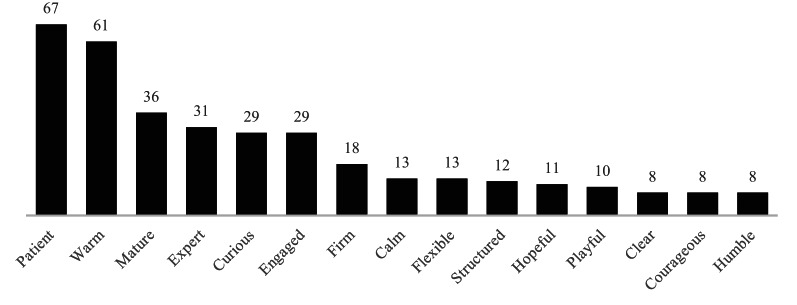
Important characteristics of health professionals working with eating disorders. Numbers on the bars indicate the number of coded responses for each characteristic (N = 137; 354 codes).

**Fig. 5:**
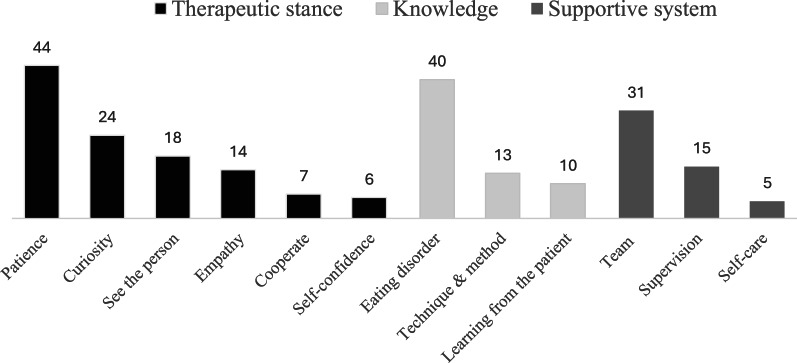
Advice to new health professionals entering the eating disorder field. Numbers on the bars (color-coded by theme) indicate the number of coded responses for each subtheme (N = 137; 227 codes).

Although formal training in qualitative methods varied among the coding team, all members brought relevant experience from both clinical work and previous involvement in research projects within specialist eating disorder treatment. This background informed their interpretation of the material and may have influenced the analytic lens applied. To further support transparency and analytic credibility, supplementary tables (S1–S5) present illustrative quotes corresponding to each theme and subtheme across the five research questions, mirroring the thematic structures shown in Figs. 1, 2, 3, 4 and 5.

## Results

Of the 188 participants, 81% were clinicians, 5% were researchers, and 14% were both clinicians and researchers. The participants represented psychologists (36.8%), nurses (14.8%), medical doctors (13.4%), dietitians (11.3%), social workers (11.3%), physiologists (4.9%), and the remaining (5.6%) fell into other professional categories. Regarding work experience in the eating disorder field, 22.5% reported 1–3 years of experience, 14% reported 4–6 years, 10.5% reported 7–10 years, 18.9% reported 11–15 years, 15.4% reported 16–20 years, and 18.2% reported more than 20 years. The following content analysis results are presented according to the five research questions. Across all questions, we report code counts to provide descriptive context; however, thematic emphasis in the results reflects the interpretive significance of patterns, not merely their frequency.

### Reasons for beginning work in the field of eating disorders

In total, 135 participants responded, generating 187 codes across nine themes (Fig. 1). For many participants, the decision to begin working in the field was an interest in the subject, which manifested in various ways. Some were drawn to the challenging nature of the work, while others were fascinated by the interplay of psychological, biological, and social factors associated with eating disorders. For a significant number, entry into the field was a matter of coincidence, driven by unexpected opportunities rather than a deliberate choice. One participant noted that it started “by coincidence, then I found it very interesting, rewarding, and stimulating in many ways, and I had the chance to learn from experienced clinicians from the start”, capturing the experience of discovering the field by chance and subsequently finding it meaningful and rewarding.

The desire to make a difference in the lives of individuals and families was another factor. This drive to help was sometimes rooted in personal connections, with some participants sharing their own experiences with eating disorders or those of someone close to them. Even without personal experience, many participants were drawn to contribute to change. To a lesser extent, participants cited treatment methods, the professional environment, or the perceived potential for professional growth.

### Challenges in working with eating disorders

A total of 168 participants responded, generating 206 codes. These codes were organized into twelve subthemes, which were further grouped under three main themes: patient-related, clinician-related, and external factors (Fig. 2). A subset of responses also described how these themes interconnect.

####  Patient factors: ambivalence and complexity

This subtheme refers to patients’ conflicting motivations in treatment, such as wanting to recover while struggling with behavioural change (e.g., weight gain, meal planning). It captures the cognitive-emotional aspects of ambivalence, along with the broader psychological and psychiatric complexity described by participants. Participants emphasized the intricate internal world of their patients. A prominent theme was ambivalence towards treatment, with conflicting desires to recover while simultaneously clinging to the eating disorder. This internal struggle is illustrated by the participant who shared that “patients are willing to leave the eating disorder but refuse to gain weight or take the appropriate steps to achieve the goal”. Participants described how this push-and-pull dynamic is further complicated by the perceived “egosyntonic” nature of the illness, where the eating disorder is intertwined with the patient’s sense of self. Participants also noted the rigidity and irrationality often associated with the eating disorder, which they expressed made it challenging to engage patients. The presence of comorbid psychiatric conditions was also highlighted by participants as challenging, adding another layer of complexity, including determining the most adequate starting point for treatment.

#### Clinician factors: emotional and relational demands

Beyond the complexities of the illness itself, participants described challenges connected to their role. A recurring theme was the difficulty of motivating and engaging patients throughout the sometimes difficult treatment process. “To work with motivation of the patient”, as one participant put it, often required sustained and repeated effort. This involved fostering a genuine therapeutic alliance and navigating patient ambivalence and skepticism. One participant noted the constant effort required to “find the motivation and hold onto it”. Another described the challenge as “establishing a therapeutic alliance with the patient”, highlighting the relational demands of the work. Other challenges related to a perceived lack of knowledge about eating disorders, including treatment approaches for many patients.

#### External factors: the system

This subtheme refers to systemic and organizational barriers that participants perceived as limiting effective treatment, including time constraints, teamwork challenges, and family-related complexity. Participants expressed frustration with the pressure to deliver effective treatment within constrained timeframes, with one noting the inadequacy of “ten sessions per patient, including both examination and treatment”. This pressure was reported to be exacerbated by a perceived lack of understanding and collaboration within the healthcare system itself, including instances of non-functioning teamwork. One explanation offered was that there were “not enough resources for good teamwork”, reflecting broader systemic challenges to interprofessional collaboration. Beyond the healthcare system, participants identified the influence of family dynamics, such as difficulties in collaborating with parents or managing complex family systems, and need to manage complex team dynamics as challenging.

#### The interplay of challenges

While categorized separately, many challenges described by participants appeared interconnected in practice. Several participants reflected on the difficulty of tailoring treatment to complex, comorbid patients within systems that prioritized symptom-based approaches. One participant noted how “rigid models of treatment” imposed by healthcare structures often clashed with the need for more flexible and individualized care. This systemic mismatch was described by some as especially burdensome, “more so than the patient herself/himself”, underscoring how organizational constraints could amplify the emotional strain of clinical work. These reflections suggest that clinicians are often required to navigate overlapping pressures across individual, relational, and organizational domains, rather than experience each challenge in isolation.

### Rewards in working with eating disorders

A total of 170 participants responded, generating 222 codes. These codes were organized into eleven subthemes, which were further grouped under three overarching themes: patient-related, clinician-related, and external factors (Fig. 3). While patient- and clinician-related factors were frequently cited, external factors were mentioned less often.

#### Patient factors: witnessing meaningful progress

This subtheme refers to the rewarding experience of observing patients improve in health, functioning, and quality of life over the course of treatment. This was one of the most frequently mentioned rewards, with many participants describing how meaningful it was to see “patients getting better”, becoming “healthy”, and reaching milestones like “gaining weight”. Participants also found rewards in observing patients cultivate a healthier relationship with food, their bodies, and their emotional landscape. As one participant stated, witnessing a patient “find a way how to relax or give up a bit” signaled a shift towards greater self-acceptance. Participants described rewards of seeing patients resume the activities that once brought them joy, includingreturning to school or work, re-engaging with hobbies, rebuilding social connections, and ultimately beginning to “live their lives in a more satisfying way” and “return to life”.

#### Clinician factors: being an integral part of the process

This subtheme refers to the professional satisfaction of playing an active role in the recovery process, particularly through building a strong therapeutic alliance. Participants highlighted the importance of building trust and fostering a confident, collaborative relationship as central to their sense of fulfillment. In this process, utilizing their skills, knowledge, and compassion was experienced as both rewarding and essential for guiding patients toward recovery. Participants expressed satisfaction in “being a part of the recovery process”, actively contributing to their patients’ journey towards health and wholeness, as illustrated by descriptions such as “help them get healthy” and “win them free of the eating disorder”.

#### External factors: family and teamwork

To a lesser extent, participants found reward in the collaborative aspects of their work, including engaging families in the treatment process and witnessing the strengthening of family bonds. One participant described the satisfaction of “experiencing parents getting more confident and get hope”, emphasizing the emotional impact of supporting families. A few participants also highlighted the rewards of functioning teamwork, emphasizing a collaborative and supportive professional environment. As one participant noted, simply, “the teamwork”, suggesting that working closely with colleagues was itself a valued part of the experience.

### Important characteristics of health professionals working with eating disorders

A total of 137 participants responded to the question, generating 354 codes categorized into 15 characteristics (Fig. 4). Participants most frequently highlighted patience as a key characteristic. While the specific understanding of this term could potentially vary, it points to approaching recovery with sensitivity to its often non-linear nature. Warmth emerged as the second most cited characteristic. Maturity, including emotional stability, self-awareness, and a grounded presence, was another frequently cited characteristic. Participants also highlighted expertise as important, following these three characteristics. In this context, expertise refers to specialized knowledge and skills related to the eating disorder itself. Less frequent characteristics highlighted by participants included curiosity, engagement, clarity in communication, and a collaborative approach.

### Advice to new health professionals entering the eating disorder field

In total, 137 participants responded, generating 227 codes across three core themes—the therapeutic stance, building and sustaining knowledge, and the supportive system—which were further divided into 12 subthemes (Fig. 5).

#### Therapeutic stance: cultivating patience, curiosity, and flexibility

This subtheme refers to participants’ emphasis on cultivating a therapeutic stance grounded in patience, curiosity, and flexibility—qualities seen as central for building a trusting relationship and tailoring care to the individual. Participants described this stance as a way of being with the patient that fundamentally shapes clinical practice, requiring acceptance of the individual, a genuine therapeutic relationship, and a collaborative, flexible approach to treatment. The most frequently mentioned advice centered around the value of patience. Participants stressed that recovery from an eating disorder is typically a long and non-linear process, advising new clinicians to “be patient, take one step at a time”. Related to patience, participants highlighted curiosity and approaching each patient with openness: “Be curious, don’t think that you know anything about this person before you start treatment”. This also extends to having a flexible approach to treatment, recognizing that “there is no one-size-fits-all treatment”. Inherent in this process is the recognition that clinical work requires dedicated time and space for reflection, and a collaborative effort to envision a future free from the eating disorder.

Participants elaborated that curiosity and flexibility are not only clinical tools but reflect a deeper therapeutic orientation: seeing the person behind the illness, understanding the psychological mechanisms sustaining it, and speaking to the patient’s hopes and dreams. At the same time, several participants emphasized the importance of accepting the starting point: that the eating disorder may currently be serving an important function for the individual, perhaps even “keeping the person alive”. This advice encourages health professionals to hold both the suffering and the potential for recovery in mind, acknowledging the complex role the eating disorder might be playing.

#### Building and sustaining knowledge

This subtheme refers to how clinicians develop and maintain the expertise and judgment needed for working in eating disorder treatment. Participants emphasized the need for a robust knowledge base, highlighting specialized training and education. Participants stressed the importance of moving beyond textbook knowledge and looking beyond surface-level symptoms: “learn to understand the eating disorder mindset”. Furthermore, participants underscored the value of “learning by doing” by taking the time to develop clinical judgment through direct patient contact, and gaining insights by listening to and understanding patients’ perspectives. This also included remaining students of the field, continuously updating professional knowledge, and staying current with research and treatment approaches.

#### The supportive system: cultivating a culture of care

This subtheme captures participants’ emphasis on the importance of support systems—including team collaboration, supervision, and personal boundaries—for clinician well-being and patient treatment. A recurring theme was the importance of working within a team. “It’s teamwork, don’t try to do it alone!” was advised by numerous participants, highlighting the challenges of facing the field in isolation. This collaborative approach, they suggested, not only benefits patients but also provides clinicians with a network of support and shared expertise.

Furthermore, participants noted the value of seeking guidance from more experienced clinicians through regular supervision. One participant suggested to “talk weekly with an experienced clinician about your patients”. Additionally, participants highlighted the importance of self-care, stressing the need for healthy boundaries between professional and personal life to promote long-term sustainability. “Don’t bring work home. We cannot save the world!” advised one participant, while another encouraged new clinicians to “take care of your relationships and make sure to spend time on things other than work and to engage in restful activities during your free time”.

## Discussion

This study investigated why health professionals specialized in eating disorders entered the field, the challenges and rewards they encounter, the characteristics they consider most important for treating patients, and their advice for newcomers to the field. A strength of this study is the diverse and experienced sample, with the majority reporting more than ten years of experience in the eating disorder field. The most common reasons for entering the field were an interest in the subject and a desire to help others, aligning with findings from other healthcare settings [20]. Furthermore, one of the most frequently reported reasons was coincidence, such as an unexpected job opportunity. The considerable experience of most participants suggests that even unplanned entries can lead to long-term engagement in the field.

The main reward for participants in our data was witnessing a positive outcome for the patient. This was sometimes described narrowly as recovery, at other times more broadly as psychological development and maturation. Rewards also included having an impact and building therapeutic relationships, and, to a lesser extent, collaborating with families and colleagues. This aligns with previous research, which has similarly identified patient recovery and the therapeutic relationships as rewarding for health professionals in the eating disorder field [11, 12]. Participants also reported a range of challenges spanning patient-related, clinician-related, and external factors, aligning with previous research [1, 2, 6, 10]. Patient ambivalence, defined as the internal struggle between maintaining the eating disorder and pursuing recovery, stood out as the most central challenge, providing further support for what has previously been highlighted in qualitative studies (e.g., 2, 7). Ambivalence is consistently identified as a defining and, in many ways, unique feature of eating disorders, and is one of the most common reasons for the high treatment dropout rates [21, 22]. The second most frequently reported challenge was the difficulty of establishing a therapeutic relationship. Notably, participants often described this challenge as closely linked to patient ambivalence, which sometimes complicates efforts to build trust and engagement. Ambivalence is a common and clinically relevant feature of therapeutic work with eating disorders [23, 24]. Recognizing the clinical relevance of ambivalence in both the disorder and treatment process may help organizations better support clinicians and improve patient engagement and outcomes [1, 25, 26].

Challenges and rewards were closely intertwined in our data. While patient ambivalence was identified as the most central challenge, our findings suggest that supporting patients through this internal conflict, even in small steps toward change, was experienced as rewarding by participants. Similarly, the satisfaction of building trust balanced the emotional demands of the therapeutic relationship. Although responses were given to separate survey questions and not linked at the individual level, these recurring patterns across responses suggest that the emotional burdens of the work may also serve as a source of meaning. Consistent with this, several participants explicitly identified the challenging nature of the work as both rewarding and as a reason for entering the eating disorder field, highlighting the sense of purpose and stimulation that comes from engaging with complex problems. Although this study did not explicitly examine the balance between challenges and rewards, our findings resonate with previous research suggesting that health professionals often find meaning in the challenges of eating disorder treatment [1, 11], and that caring for individuals in need can be deeply rewarding [27, 28]. This interpretation aligns with occupational health models suggesting that demanding work, when met with sufficient support and resources, can foster engagement and resilience, whereas imbalances increase the risk of strain and burnout [29, 30]. In this context, organizational resources such as supervision, team collaboration, and formalized support structures may help transform challenges into a sense of professional purpose [25]. Thus, in the right context, the very challenges of this work may become its greatest rewards.

Regarding important characteristics for health professionals working with eating disorders, our findings highlight the importance of patience and warmth, and of possessing sufficient maturity and expertise. While previous research has similarly emphasized warmth and expertise [3, 15, 16], our results suggest that patience—understood as the ability to adapt to the often slow, non-linear nature of recovery and to respond constructively to patient ambivalence—is especially crucial. This emphasis is perhaps unsurprising, considering that many patients remain ill and ambivalent toward recovery even after several years of treatment [31]. Closely related to patience, participants also emphasized the importance of a therapeutic stance characterized by openness, curiosity, and approaching each patient without preconceived notions, consistent with longstanding therapeutic ideas, such as *the constructive use of ignorance* [32] and *the not-knowing position* [33].

The ability to develop and maintain the above-mentioned therapeutic stance is, at least partly, shaped and constrained by organizational priorities and policies [34]. Although prioritizing short-term efficiency, such as rapid symptom reduction and standardized protocols, can be highly effective for some patients with eating disorders, a substantial proportion of patients require a more individualized pace or approach to treatment [31]. An exclusive organizational focus on short-term efficiency may therefore limit health professionals’ capacity to meet these diverse needs [35], and may lead to increased dropout rates from treatment and lower overall success rates [21]. This is particularly relevant when working with patient ambivalence, which often necessitates a prolonged and non-linear therapeutic process [31, 33]. Without sufficient time and structural support, clinicians may find it harder to remain engaged in the therapeutic process in the face of slow or fluctuating progress. As a result, patient ambivalence may increasingly be perceived not as part of recovery, but as resistance or low motivation, potentially leading to premature termination rather than continued engagement [1, 24]. This aligns with broader research on emotional labour in healthcare, which shows that constrained resources and organizational pressure can reduce clinicians’ emotional availability and engagement in demanding therapeutic work [36]. Ironically, this may diminish treatment effectiveness and prolong illness, and thereby undermining the very efficiency that healthcare systems seek to achieve [37]. Additionally, health professionals may experience ethical distress, reduced control, and increased job strain [38], which are well-known risk factors for job dissatisfaction and burnout [39]. These factors contribute to staff turnover and subsequent loss of expertise (e.g., [25]), which can compromise patient outcomes [26] and have financial consequences for healthcare organizations [40].

Expertise was another dominant theme in advice to newcomers. Clinical guidelines and patient perspectives agree on the necessity of specialized knowledge and ongoing training [3, 14, 16]. Expertise develops over time, through experience, feedback, and reflection, and is best supported by organizations that prioritize professional development, access to supervision, and a culture of learning [41]. In parallel, the cultivation of characteristics such as patience, empathy, and expertise also depends on the organizational context, as supportive organizational cultures tend to enhance these characteristics, while unsupportive environments tend to promote the opposite [42]. Furthermore, targeted professional training may enhance important characteristics, such as empathy [43].

Finally, our findings underscore the importance of collegial support, supervision, and a positive workplace climate in sustaining professional well-being. Consistent with leading occupational health models, social support from colleagues, supervisors, and organizations buffers the demands of emotionally demanding work and enables long-term engagement [29, 30]. In its absence, health professionals become more vulnerable to stress, detachment, and reduced quality of care. Professional environments that prioritize collaboration and support not only foster staff well-being but also improve patient outcomes [4, 29], highlighting the mutual dependence of clinician sustainability and quality of care.

### Clinical implications

This study has several practical implications for clinical work, both for individual clinicians and for organizations. For clinicians, it is important to recognize that eating disorder treatment often involves slow progress, emotional intensity, and relational complexity. A professional stance of patience, warmth and curiosity combined with specialist knowledge of eating disorders and common comorbidities appear particularly relevant.

For healthcare organizations, the findings emphasize the need to actively support clinical reality. Treatment environments should enable relational work by ensuring time for therapeutic engagement, access to supportive team structures and regular supervision, and opportunities for ongoing training. When these conditions are in place, the emotional demands of the work may not only be more manageable but can also become a source of meaning and professional satisfaction.

### Limitations

Several limitations should be considered. The predominance of psychologists, along with limited demographic and workplace information, may limit the representativeness and generalizability of the findings; however, the multidisciplinary sample provides a broader perspective compared to previous research. Most participants had more than ten years of experience in the eating disorder field, a strength in terms of clinical wisdom and expertise, but the findings may not fully capture the perspectives of early-career professionals or non-psychologists, whose responses may differ from those who have remained in the field long-term. In addition, participation in the study was voluntary, which may have introduced self-selection bias. Many responses, especially those regarding professional characteristics, were brief and limited to single adjectives, reducing analytical depth and allowing for varied interpretations. While themes and subthemes were treated as distinct, some conceptual overlap occurred due to the multifaceted nature of responses. These cases were resolved through coder consensus to ensure clarity and consistency. Self-reported data introduce risks of recall and social desirability bias, although responses were anonymous. The content analysis was conducted by researchers who are also practicing clinicians in the eating disorder field. While this background may enhance the relevance and contextual sensitivity of the analysis, it may also have shaped the interpretive lens, despite efforts to ensure credibility through independent coding and consensus procedures. As with any hermeneutic process, interpretation is inevitably shaped by the researchers’ preunderstandings and the co-construction of meaning through interaction with the data. Responses were submitted in several Nordic languages and English. Google Translate was used for Icelandic, and for Finnish except by one fluent coder. Full coder agreement supported analytic credibility and mitigated risks associated with translation. Nonetheless, some nuances may have been lost, particularly for culturally situated or idiomatic expressions that may not translate seamlessly across Nordic contexts. Translations were cross-checked through coder consensus, but no formal back-translation was conducted. Finally, the cross-sectional survey design precludes conclusions about change over time or causality.

### Implications for future research

Future studies could examine the perspectives of those who have recently entered or left the field, or who have lived experience of eating disorders, to clarify drivers of retention and attrition; test how the interplay between challenges and rewards relates to clinician well-being and patient outcomes; evaluate training approaches to cultivate clinician characteristics and a patient, curious therapeutic stance; and identify organizational conditions (e.g., supervision structures, protected time, team composition) that enable these practices in day-to-day care.

## Conclusions

Challenges and rewards in eating disorder services are intertwined for health professionals: patient ambivalence and the emotional work of building trust are central challenges, while supporting meaningful steps toward change is a common source of reward. In this context, patience, warmth, maturity, and specialist knowledge of eating disorders emerged as key professional characteristics, and practical advice emphasized a patient and curious approach, continued learning, and reliance on supervision and teamwork. Taken together, these findings underscore the need to cultivate these characteristics within supportive organizational contexts. In particular, services can design training that develops both relational skills and specialized eating disorder knowledge, provide regular supervision, and protect time and pacing for treatment so health professionals can navigate the often non-linear course of recovery. These practice conditions can inform workforce development, training curricula, and service design to support staff retention and, in turn, high-quality care.

## Supplementary Information

Below is the link to the electronic supplementary material.

Supplementary Material 1

## Data Availability

De-identified materials (codebook, aggregated code frequencies, and anonymized excerpts) are available from the corresponding author on reasonable request, subject to institutional approvals; full raw qualitative responses cannot be shared publicly to protect confidentiality.
