# Supplementary material for: Experts’ wisdom: challenges, rewards, key clinician characteristics, and advice in eating disorder treatment
Source: J Eat Disord. 2025 Oct 27;13:239. doi: 10.1186/s40337-025-01438-0 (PMC12560607; doi:10.1186/s40337-025-01438-0)
Supplement: Supplementary file 1 — Supplementary Material 1 [file 40337_2025_1438_MOESM1_ESM.pdf]

Supplementary Table 1. Reasons for beginning work in the field of eating disorders

Theme	Example Extract
Interesting	– Interesting<br>– Desire to understand the disorder, cause, mechanism, way out
Coincidence	– My manager asked me to join a regional work-group<br>– It is part of my work, I did not decide
Desire to help others	– I feel i can make a difference in someone's life<br>– Possible to make a big difference
Challenging	– Challenging<br>– Difficult to help the patients
Enjoyable	– It's rewarding<br>– Exciting
Treatment method	– Family therapy focus<br>– You can work with therapy
Personal experience	– Had AN myself<br>– Many friends developed EDs
Patient characteristics	– I think these are patients with a lot of resources<br>– The patients are wise
Professional environment	– Good professional environment<br>– Interdisciplinarity
Unsure	– Unsure

Supplementary Table 2. Challenges in working with eating disorders

Theme (Main)	Subtheme	Example Extract
Patient-related factors	Ambivalence	– Ambivalence towards recovery<br>– Ambivalence towards treatment
	Psychology	– Ego syntonic<br>– The exceptionally poor interoceptive awareness
	Eating disorder diagnosis	– The complexity of eating disorders<br>– Anorexia
	Comorbidity	– Patients suffer from more than one problem, it's hard to know what problem to start with<br>– Somatic complications and psychiatric comorbidity
	Emotions	– Lack of hope<br>– Shame
Clinician-related factors	The relationship	– Building trust<br>– To endure the long time of no advance in treatment, to avoid giving up on the patients' behalf
	Knowledge	– Difficult to understand the cause and maintenance<br>– How to meet them the right way
	Method	– Lack of evidence for treatment<br>– Offer efficient treatment
External factors	Family	– Help the family to handle the anxiety<br>– Get parents on board for good enough routines
	Healthcare system	– No backup from the organization<br>– The lack of collaboration between somatic and psychiatric care
	Resources	– Not enough resources for good teamwork<br>– The time aspect, often not enough to meet needs.
	Teamwork	– The challenge of good teamwork<br>– Consistency within treatment team

Note. This table mirrors the thematic structure presented in Figure 2.

Supplementary Table 3. Rewards in working with eating disorders

Theme (Main)	Subtheme	Example Extract
Patient-related factors	Outcome	– When they get better<br>– When patients start to eat
	Psychological development	– The process of patients starting to become curious of themselves, openly exploring who they are, what they feel, who they want to be<br>– That change can occur on a deep level
	Back to life	– To see recovery and how patients with good resources can begin to live meaningful lives again, even after very serious functional decline related to the disease<br>– Patients resuming their normal lives
	Motivation	– When the patient shows that she/ he really want to make changes<br>– When they can turn and start working to be well
Clinician-related factors	Making a difference	– The possibilities to make a real big difference for the patients and their families<br>– To guide patients to find and experience signals and information from the body again
	The relationship	– When you experience a good relationship with the patient and feel that you are working towards the same goal<br>– The opportunities to earn patients' trust
	Challenge	– To have to think a lot<br>– Challenging
	Knowledge	– Desire to use my knowledge of the subject<br>– Gaining new clinical relevant knowledge through research
	Method	– Good treatment models<br>– Working with family oriented treatment
External factors	Family	– When you succeed in supporting the family to overcome ambivalence and move forward as a whole and strengthened family<br>– Help the parents to handle the anxiety as a family project
	Teamwork	– Working in a team<br>– Collaborative teamwork

Note. This table mirrors the thematic structure presented in Figure 3.

Supplementary Table 4. Important characteristics of professionals working with eating disorders

Theme	Example Extract
Patient	– Patience<br>– Perseverance
Warm	– Empathic<br>– Warm<br>– Supportive
Mature	– Stability<br>– Mature<br>– Mentalizing
Expert	– Experience<br>– Both somatic and psychiatric competence
Curious	– Listen<br>– Curious<br>– Interested
Engaged	– Active<br>– Engaged in their patients
Firm	– Assertive in a kind way<br>– Challenging
Calm	– Calm<br>– Relaxed
Flexible	– Every patient is different<br>– Flexible
Structured	– Follow the plan<br>– Structured
Hopeful	– Keep hope<br>– Have faith<br>– Optimism
Playful	– Playful<br>– Good sense of humor<br>– Creative
Clear	– Clear<br>– Honest
Courageous	– Be brave<br>– Have courage
Humble	– Thank your patients<br>– Humility regarding parents' situation

Supplementary Table 5. Advice to new professionals entering the eating disorder field

Theme (Main)	Subtheme	Example Extract
Therapeutic stance	Patience	– Be patient<br>– Remember that it takes time for the person to change
	Curiosity	– Be open-minded<br>– Listen to what they really are saying, instead of formulating your answer before they have finished
	See the person	– See the person behind the symptom<br>– See your patient's strengths
	Empathy	– Empathy<br>– Kindness
	Cooperate	– Cooperate with the patient;<br>– Make the relationship work, then you can hit the ED
	Self-confidence	– Do not be afraid to set limits or say no if needed<br>– Don't be afraid to make mistakes
Knowledge	Eating disorder	– Learn a lot about ED<br>– Get to know the field. Theory and practice<br>– Take the time to understand why treatment non-negotiables are important
	Technique & method	– Food diary<br>– Keep to the plan
	Learning from the patient	– Learn from your patients<br>– Learning by doing
Supportive system	Team	– Don't work alone<br>– Talk to your colleagues.
	Supervision	– Seek guidance<br>– Seek information and guidance from others who have more experience in the field
	Self-care	– Have a personal life, not just work<br>– Don't take your work home. We can't save the world!

Note. This table mirrors the thematic structure presented in Figure 5.
